# Vision-Based Hybrid Controller to Release a 4-DOF Parallel Robot from a Type II Singularity

**DOI:** 10.3390/s21124080

**Published:** 2021-06-13

**Authors:** José L. Pulloquinga, Rafael J. Escarabajal, Jesús Ferrándiz, Marina Vallés, Vicente Mata, Mónica Urízar

**Affiliations:** 1 Departamento de Ingeniería de Sistemas y Automática, Instituto de Automática e Informática Industrial, Universitat Politècnica de València, 46022 Valencia, Spain; raessan2@etsii.upv.es (R.J.E.); jeferal@etsid.upv.es (J.F.); mvalles@isa.upv.es (M.V.); 2 Departamento de Ingeniería Mecánica y de Materiales, Instituto Universitario de Investigación Concertado de Ingeniería Mecánica y Biomecánica, Universitat Politècnica de València, 46022 Valencia, Spain; vmata@mcm.upv.es; 3 Department of Mechanical Engineering, Faculty of Engineering in Bilbao, University of the Basque Country, 48013 Bilbao, Spain; monica.urizar@ehu.es

**Keywords:** singular configuration, parallel robot, motion control, 3D tracking, screw theory

## Abstract

The high accuracy and dynamic performance of parallel robots (PRs) make them suitable to ensure safe operation in human–robot interaction. However, these advantages come at the expense of a reduced workspace and the possible appearance of type II singularities. The latter is due to the loss of control of the PR and requires further analysis to keep the stiffness of the PR even after a singular configuration is reached. All or a subset of the limbs could be responsible for a type II singularity, and they can be detected by using the angle between two output twist screws (OTSs). However, this angle has not been applied in control because it requires an accurate measure of the pose of the PR. This paper proposes a new hybrid controller to release a 4-DOF PR from a type II singularity based on a real time vision system. The vision system data are used to automatically readapt the configuration of the PR by moving the limbs identified by the angle between two OTSs. This controller is intended for a knee rehabilitation PR, and the results show how this release is accomplished with smooth controlled movements where the patient’s safety is not compromised.

## 1. Introduction

Parallel robots (PRs) are composed of two or more closed kinematic chains connecting a fixed and a mobile platform that defines the end-effector to be controlled [1]. As opposed to their serial counterpart, they benefit from greater accuracy, stiffness, and load capacity, making them suitable for a great variety of applications [2,3]. Human–robot interaction is one of the major applications, for instance, in the context of medical rehabilitation [4]. Within this field, lower limb rehabilitation [5,6,7,8,9] is an active research area. However, PRs also present several drawbacks regarding the size of their workspace and the presence of singularities within the workspace. The former can be addressed by means of a proper mechanical design of the PR to cover the workspace as required, while the latter requires further analysis.

Singularities in a PR were first analysed by Gosselin and Angeles [10], who established a classification of singular configurations according to the characteristics of the Jacobian matrices calculated from constraint equations. They defined a type I (or inverse kinematic) singularity to refer to the loss of at least one degree of freedom (DOF) due to a degeneracy of the inverse Jacobian matrix ($\|J_{I}\|=0$) and a type II (or forward kinematic) singularity to indicate the gain of at least one DOF caused by the degeneracy of the forward Jacobian matrix ($\|J_{D}\|=0$). Some other related classifications of singular configurations can be found in [11,12]. type I singularities typically occur as the manipulator approaches the boundary of the workspace and are easy to detect and avoid, but type II singularities can arise within the workspace and are more difficult to treat [13].

Type II singularities prevent the mobile platform from bearing external forces despite having all the actuators locked, leading to an uncontrolled motion of the end-effector. The main goal of lower-limb rehabilitation is to perform specific movements that stimulate the motor plasticity of the patient to improve the motor recovery [5]. In conventional rehabilitation, the movements of the patient are controlled and monitored by a physiotherapist, while in robotic rehabilitation, the control task is performed by a PR. For this task, a PR must ensure stiff behaviour despite the presence of type II singularities to maintain control during the rehabilitation process.

Extensive research has been conducted to tackle type II singularities. The determinant of the forward Jacobian $J_{D}$ gives no further information than the proximity to a singularity, as it lacks a physical meaning [14]. Based on screw theory [15], a transmission index (TI) was designed by Yuan et al. [16] to express the quality of force and motion transmission by using the transmission wrench screw (TWS) and output twist screw (OTS). Takeda and Funabashi [17] designed a TI that expresses how each actuator individually contributes to the motion of the mobile platform by leaving just one actuator active and the rest locked. Subsequently, Wang et al. [18] using the TI proposed by Takeda and Funabashi established that for a type II singularity, at least two OTSs are linearly dependent. Pulloquinga et al. [19] proposed the angle between two instantaneous screw axes from the OTSs (Ω) as a measure for the proximity detection of type II singularities, providing physical meaning and the capability to determine the chains producing the singular configuration.

The extensive analysis of type II singularities presented has been incorporated in motion/force performance evaluation [18,20], path planning, and the design of reconfigurable PRs [21,22]. These analyses have been developed offline, and very little has been found about including this information in the control unit of the PR [23,24]. Abgarwal et al. [24] designed a control scheme to avoid type II singularities of a planar PR by using artificial potential functions. The potential functions are activated near the singularity to alter the trajectory by means of repulsion forces. This setting prevents the PR from entering into a singular configuration by avoiding it. However, an evader controller cannot deal with the situation in which the robot is initially in a type II singularity. Such a task would require extra instrumentation, since solving the forward kinematic problem based on the joint variable measures does not have a single solution. The various possible positions of the mobile platform are due to the degeneracy of the forward Jacobian matrix.

One unambiguous solution to estimate the actual pose of the PR is by using a vision system [25]. Huynh et al. [26] implemented a vision/position hybrid control for a Hexa PR by defining a two-level closed-loop controller. Amarasinghe et al. [27] designed a vision-based hybrid control on a mobile robot. It could autonomously reach a docking station by using a finite-state machine and proportional control combined with image processing.

However, to the best of the authors’ knowledge, no research has been published focusing on PR singularity releasing, i.e., letting the robot autonomously get out of a type II singularity. In this paper, a novel algorithm based on online readings from a vision system is proposed to release the PR from a type II singularity. The proposed algorithm is the first to use the angle Ω as an online detector for the proximity to singular configurations. This algorithm is integrated into a two-level closed-loop hybrid controller that results in more compliant manipulation when performing knee rehabilitation tasks. In the inner loop, there is an algebraic closed-loop controller. The outer loop implements a vision-based controller whose algorithm determines the two limbs that most affect the type II singularity by means of the angle Ω. Then, only the references of those two limbs are modified online to feed the inner loop of the controller.

Section 2 describes the 4-DOF PR for knee rehabilitation used to perform the simulations and experiments. Next, the mathematical foundations of type II singularities and the angle Ω are explained. Then, the 3D vision system that has been used to keep track of the pose of the PR is presented, together with a detailed description of the proposed vision-based hybrid controller. Section 3 begins with a description of the requirements for simulation and experimentation as well as the singular trajectories that were designed for this research. The main results are also presented in this section. Finally, the results are discussed in Section 4.

## 2. Materials and Methods

This section presents the mathematical foundation used in the development of the angle Ω that detects the proximity to a type II singularity in a knee rehabilitation PR. Subsequently, the 3D tracking system (3DTS) used to measure the actual pose of the mobile platform is described. Then the 3DTS and Ω are combined to develop a novel vision-based hybrid controller to release the actual PR under study from a type II singularity. This section also includes a detailed explanation of the algorithm corresponding to this hybrid controller, which detects and moves the actuators according to the angle Ω.

### 2.1. 3UPS+RPU Parallel Robot

**Figure 1:**
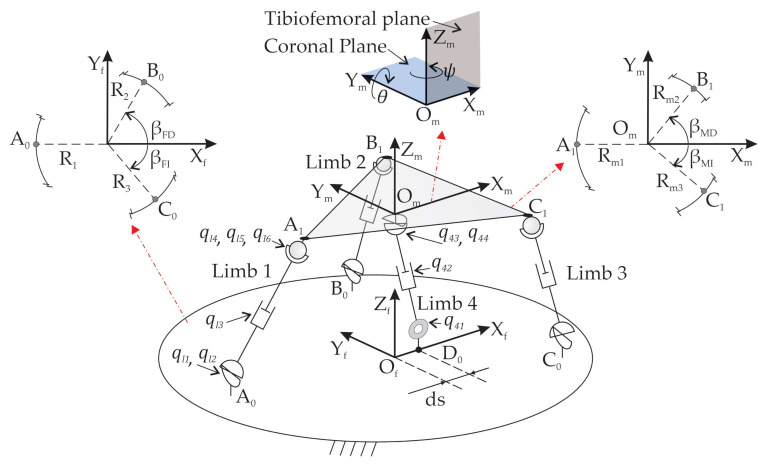
Mechanical configuration of the 3UPS+RPU PR.

After knee surgery, the diagnosis and rehabilitation tasks require two translational movements ($x_{m}$, $z_{m}$) in the tibiofemoral plane, one rotation (ψ) around the coronal plane and one rotation (θ) around the tibiofemoral plane [28]. These four DOFs are shown in Figure 1. In order to accomplish these requirements, a PR with 4-DOF has been designed, built [29], and optimized [30] at the Universitat Politècnica de València. The PR under study is named 3UPS+RPU due to its four-limb architecture. The external limbs or open kinematic chains have a UPS configuration, while the central one has an RPU configuration (see Figure 1). The letters R, U, S and P stand for revolute, universal, spherical and prismatic joints, respectively, and the actuated joints are indicated by the underlined format.

The kinematic model of the 3UPS+RPU PR is established by 15 generalized coordinates as follows:

- The position ($x_{m}$, $z_{m}$) and the orientation (θ, ψ) of the mobile platform.
- The orientation of the four universal joints: $q_{l1}$, $q_{l2}$ for limbs l=1…3 and $q_{43}$, $q_{44}$ for limb 4.
- The length of the four linear actuators given by $q_{l3}$ for limbs l=1…3 and $q_{42}$ for limb 4.
- The orientation of the three spherical joints represented by $q_{l4}$, $q_{l5}$, $q_{l6}$ for external limbs l=1…3.
- The orientation of the revolute joint $q_{41}$.

The variables q are measured with respect to a local reference system attached to each joint. The coordinates $x_{m}$, $z_{m}$, θ, and ψ are measured with respect to the reference system $\left\{O_{f}-X_{F}Y_{F}Z_{F}\right\}$ attached to the centre of the fixed platform to reduce the complexity of the model.

The locations of $A_{0}$, $B_{0}$, $C_{0}$, and $D_{0}$ that link the four limbs to the fixed platform are defined by the geometric variables $R_{1}$, $R_{2}$, $R_{3}$, $\beta_{FD}$, $\beta_{FI}$, and $d_{s}$. The locations of $A_{1}$, $B_{1}$, $C_{1}$, and $O_{m}$ that link the limbs to the mobile platform are defined by the geometric variables $R_{m1}$, $R_{m2}$, $R_{m3}$, $\beta_{MD}$, and $\beta_{MI}$. Figure 1 shows the location of $A_{0}$, $B_{0}$, and $C_{0}$ and $A_{1}$, $B_{1}$, and $C_{1}$ on the fixed and mobile platform. Table 1 shows the values of $R_{1}$, $R_{2}$, $R_{3}$, $\beta_{FD}$, $\beta_{FI}$, and $d_{s}$ measured with respect to $\left\{O_{f}-X_{F}Y_{F}Z_{F}\right\}$ and $R_{m1}$, $R_{m2}$, $R_{m3}$, $\beta_{MD}$, and $\beta_{MI}$ with respect to $\left\{O_{m}-X_{M}Y_{M}Z_{M}\right\}$. The mobile reference system $\left\{O_{m}-X_{M}Y_{M}Z_{M}\right\}$ is attached to the centre of the mobile platform.

### 2.2. Type II Singularities

The velocity equations of a PR [10] are defined by the time derivatives of the geometrical constraint equations ($\vec{\Phi }$) as follows:

(1) $$J_{D}\dot{\vec{X}}+J_{I}{\dot{\vec{q}}}_{ind}=\vec{0}$$

$\vec{X}$ is the set of outputs that represents the DOFs of the mobile platform; ${\vec{q}}_{ind}$ is the set of inputs that corresponds to the active joint length; and $J_{D}$ and $J_{I}$ are the forward and inverse Jacobian matrices, respectively. $J_{D}$ and $J_{I}$ are FxF square matrices for a non-redundant PR, where F is the number of DOF.

A type II singularity takes place when $J_{D}$ is rank deficient; i.e., its determinant is zero ($\|J_{D}\|=0$). In this configuration, if an external force is applied to the PR, the mobile platform may move ($\dot{\vec{X}}\neq 0$) despite the actuators being locked (${\dot{\vec{q}}}_{ind}=0$). For this reason, in a type II singularity, the control over the PR is lost, becoming potentially dangerous for the user or the robot itself. The PR under study must ensure a safe interaction with the knee of the patient, and, therefore, the treatment of type II singularities is an important problem to solve. A general method to detect the proximity to a type II singularity is by calculating the $\left\|J_{D}\right\|$.

In the 3UPS+RPU PR, $J_{D}$ is defined as a 4×4 matrix,

(2) $$J_{D}=\left[\frac{\partial \vec{\Phi }}{\partial x_{m}}\;\;\frac{\partial \vec{\Phi }}{\partial z_{m}}\;\;\frac{\partial \vec{\Phi }}{\partial \theta }\;\;\frac{\partial \vec{\Phi }}{\partial \psi }\right]$$

with $\vec{X}={\left[x_{m}\;\;z_{m}\;\;\theta \;\;\psi \right]}^{T}$ and ${\vec{q}}_{ind}={\left[q_{13}\;\;q_{23}\;\;q_{33}\;\;q_{42}\right]}^{T}$.

The online calculation of $J_{D}$ requires an accurate measure of $\vec{X}$. In a model-based controller, $\vec{X}$ is estimated by solving the forward kinematic problem using the sensors installed in the actuated joints. In a type II singularity, the forward kinematic problem presents several feasible solutions, and an unambiguous estimation of the actual $\vec{X}$ is not possible. The accurate measure of the actual $\vec{X}$ of the PR requires direct sensing of the mobile platform by means of extra instrumentation, such as a 3DTS.

### 2.3. Angle between Two Output Twist Screws

The motion of the mobile platform of a PR is produced by the combined action of the active joints, making it difficult to identify the contribution of each actuator. Takeda and Funabashi [17] divided the movement of the mobile platform ($) into F OTSs as follows:

(3) $$\$=\rho_{1}{\hat{\$}}_{O}{}_{1}+\rho_{2}{\hat{\$}}_{O}{}_{2}+\ldots +\rho_{F}{\hat{\$}}_{O}{}_{F}$$

with

(4) $${\hat{\$}}_{O}=\left({\vec{\mu_{\omega }}}_{O};{\vec{\mu_{v}}}_{O}^{*}\right)$$

$\rho_{i}$ is the amplitude for each OTS, and ${\vec{\mu_{\omega }}}_{O}$ and ${\vec{\mu_{v}}}_{O}^{*}$ are the instantaneous screw axis and the linear component of the normalized OTS (${\hat{\$}}_{O}$), respectively. Each ${\hat{\$}}_{O}$ is determined by solving Equation (5) considering that F−1 actuators are locked.

(5) $${\hat{\$}}_{O}{}_{i}\circ {\hat{\$}}_{T}{}_{j}=0\;\;\left(i,j=1,2,\ldots ,F,\;i\neq j\right)$$

where ∘ stands for the reciprocal product, and ${\;\hat{\$}}_{T}$ is the unitary TWS.

In [18], Wang et al. proved that for a singular configuration of a PR, at least two ${\hat{\$}}_{O}$s are linearly dependent. This means that in a type II singularity, both ${\vec{\mu_{\omega }}}_{O}$ and ${\vec{\mu_{v}}}_{O}^{*}$ are equal or parallel. Based on this feature, a novel type II singularity proximity index is defined by measuring the angle $\Omega_{i,j}$ between two ${\vec{\mu_{\omega }}}_{O}$s and verifying the equality of their respective ${\vec{\mu_{v}}}_{O}^{*}$. Grouping in pairs the F ${\vec{\mu_{\omega }}}_{O}$, there are $\left(\begin{array}{c} F\\ 2 \end{array}\right)$ angles Ω, which are defined as:

(6) $$\Omega_{i,j}=acos\left({\vec{\mu_{\omega }}}_{O_{i}}\cdot {\vec{\mu_{\omega }}}_{O_{j}}\right)\;\;\left(i,j=1,2,\ldots ,F,\;i\neq j\right)$$

where i and j represent the selected ${\vec{\mu_{\omega }}}_{O}$.

In contrast with the $\left\|J_{D}\right\|$, the index $\Omega_{i,j}$ provides a physical scale for the measure of the proximity to a type II singularity. When $\Omega_{i,j}=0$ and ${\vec{\mu_{v}}}_{O_{i}}^{*}={\vec{\mu_{v}}}_{O_{j}}^{*}$, ${\hat{\$}}_{O}{}_{i}$ and ${\hat{\$}}_{O}{}_{j}$ are linearly dependent in Equation (3), identifying the open chains (i, j) involved in the type II singularity. Considering the centre of the mobile platform of the 3UPS+RPU PR, six possible $\Omega_{i,j}$s are considered:

(7) $$\begin{array}{cc} \Omega_{1,2}=\arccos\left({\vec{\mu_{\omega }}}_{O_{1}}\cdot {\vec{\mu_{\omega }}}_{O_{2}}\right) & \Omega_{2,3}=\arccos\left({\vec{\mu_{\omega }}}_{O_{2}}\cdot {\vec{\mu_{\omega }}}_{O_{3}}\right)\\ \Omega_{1,3}=\arccos\left({\vec{\mu_{\omega }}}_{O_{1}}\cdot {\vec{\mu_{\omega }}}_{O_{3}}\right) & \Omega_{2,4}=\arccos\left({\vec{\mu_{\omega }}}_{O_{2}}\cdot {\vec{\mu_{\omega }}}_{O_{4}}\right)\\ \Omega_{1,4}=\arccos\left({\vec{\mu_{\omega }}}_{O_{1}}\cdot {\vec{\mu_{\omega }}}_{O_{4}}\right) & \Omega_{3,4}=\arccos\left({\vec{\mu_{\omega }}}_{O_{3}}\cdot {\vec{\mu_{\omega }}}_{O_{4}}\right) \end{array}$$

**Figure 2:**
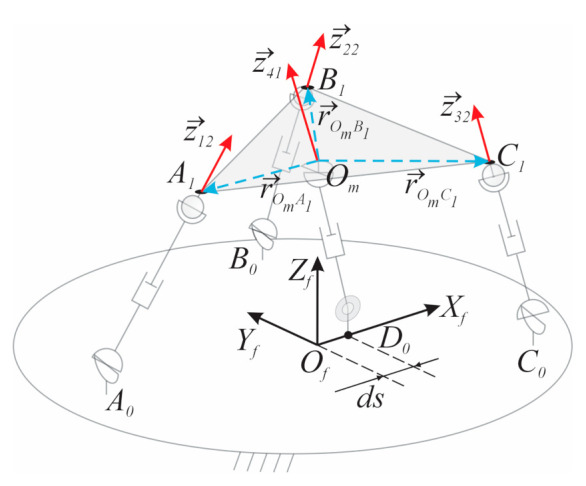
TWSs in the 3UPS+RPU PR.

The variables ${\vec{\mu_{\omega }}}_{O_{1}}\ldots {\vec{\mu_{\omega }}}_{O_{4}}$ are calculated by solving Equation (5) with the four ${\hat{\$}}_{T}$s of the linear actuators defined as

(8) $${\hat{\$}}_{T}{}_{1}=\left[\begin{array}{c} {\vec{z}}_{12}\\ {\vec{r}}_{O_{m}A_{1}}\times {\vec{z}}_{12} \end{array}\right],\;{\hat{\$}}_{T}{}_{2}=\left[\begin{array}{c} {\vec{z}}_{22}\\ {\vec{r}}_{O_{m}B_{1}}\times {\vec{z}}_{22} \end{array}\right],{\hat{\$}}_{T}{}_{3}=\left[\begin{array}{c} {\vec{z}}_{32}\\ {\vec{r}}_{O_{m}C_{1}}\times {\vec{z}}_{32} \end{array}\right],{\hat{\$}}_{T}{}_{4}=\left[\begin{array}{c} {\vec{z}}_{41}\\ \vec{0} \end{array}\right]$$

where ${\vec{z}}_{\;}$ is the unit vector of the forces applied by the actuators, and $\vec{r}$ is the position vector for the connection point of the limbs with the mobile platform measured from $O_{m}$; see Figure 2.

The capability to detect the proximity to a type II singularity given by the six $\Omega_{i,j}$ indices defined in Equation (7) has been verified from an analytical and experimental perspective [19]. However, the capability to identify the pair of limbs responsible for the type II singularity has not been exploited. Therefore, this study proposes a novel hybrid controller that takes advantage of the index $\Omega_{i,j}$ to release the 3UPS+RPU PR from a type II singular configuration. The index $\Omega_{i,j}$ is defined by means of the position and orientation of the mobile platform. For this reason, an accurate measure of the actual $\vec{X}$ is essential for developing the hybrid controller proposed.

### 2.4. 3D Tracking System

To be able to capture the movements of the mobile platform of the PR, a 3D tracking system (3DTS) based on artificial vision was used. The system consists of 10 Flex13 cameras from the manufacturer OptiTrack (Corvallis, OR, USA). These cameras use the infrared emission principle to be able to capture and detect the reflection that it creates on markers made of reflective 3M material.

**Figure 3:**
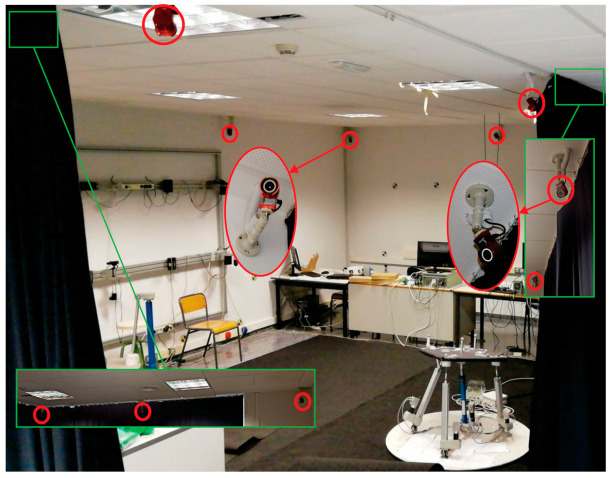
Robotics Laboratory equipped with the OptiTrack 3DTS.

Figure 3 shows the Robotics Laboratory and some cameras of the 3DTS used in this work. The cameras have a 1.3 Megapixel resolution and a capture velocity of 120 Hz. They have a latency or frame delay of 8.3 ms. The set of 10 cameras and the use of high-quality 14 mm markers make it possible to obtain an accuracy of more than 0.1 mm.

**Figure 4:**
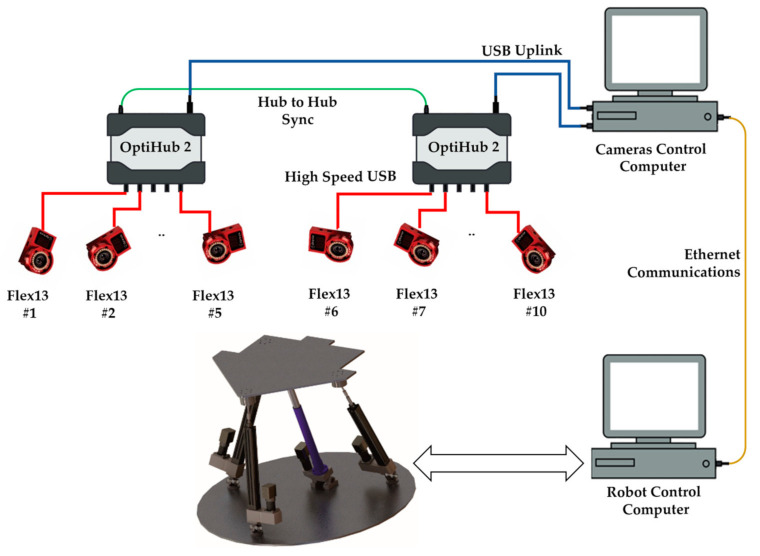
Laboratory OptiTrack 3DTS architecture.

The cameras are connected to two OptiHub2 devices. The OptiHub2 allows higher and more consistent power delivery to cameras for enhanced tracking range, simpler camera setup and cabling, and support for camera synchronization. The OptiHub2 devices are connected to high-speed USB ports in the camera control computer, and this computer communicates with the robot control computer using an Ethernet connection. The Figure 4 below shows the architecture of the OptiTrack 3DTS of the laboratory.

The Motive Tracker software (Motive) from the same manufacturer, OptiTrack, is used on the camera control computer. This software is used to perform vision system calibration and obtain 6-DOF positioning results of objects within the tracking area. Motive uses high-level tracking filters and constraints to fine tune the performance of the high-speed object tracking. Motive associates a custom set of markers to a virtual element called rigid body and offers data access at any stage in the pipeline, i.e., 2D camera images, marker centroid data, labelled markers, and rigid bodies. In addition, it is possible to completely replace the Motive user interface and directly control the system operation in a new application with the NatNet SDK.

**Figure 5:**
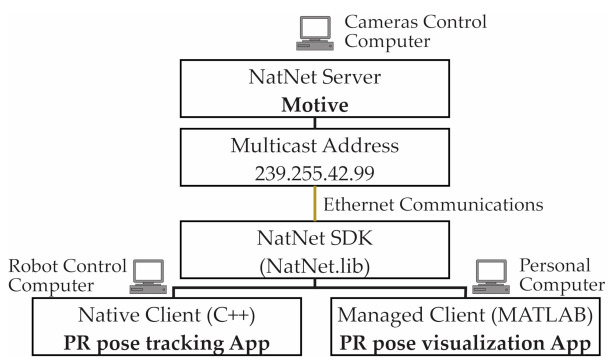
Software architecture of the OptiTrack 3DTS.

NatNet’s client/server architecture allows client applications to run on the same system as the tracking software (Motive), on separate system(s), or both. The SDK integrates seamlessly with standard APIs (C/C++/.NET), tools (Microsoft Visual Studio), and protocols (UDP/Unicast/Multicast). Using the NatNet SDK, developers can quickly integrate OptiTrack motion tracking data into new and existing applications, including custom plugins for third-party applications and engines for real-time streaming. In addition, this SDK provides a .NET interface and sample programs that work directly with MATLAB core, requiring no additional MATLAB modules. Figure 5 summarizes the software architecture of the 3DTS used in this study.

Regarding the experimental setup, each camera individually builds a 2D image based on the markers’ location, so a calibration process is required prior to the experiments in order to ensure that the system correctly reconstructs the 3D position of every marker.

The first step involved in this process is the correct orientation of the cameras to aim them at the workspace and, specifically, at the tracking volume, which, in this case, is the 4-DOF PR. Since the robot always operates in the same location and its workspace is limited, no changes in the camera location or orientation are required, and, therefore, they remain in the same position from the moment they are installed.

Another aspect to control is the brightness and illumination of the scene, as this allows the markers to be visible for the cameras, and, as such, no other unwanted objects are detected. Since the lighting conditions are the same for all experiments, some configuration parameters of the cameras, such as the exposure time, the gain, and the threshold, are set at constant values for all cameras using the software. If any intrusive markers are detected, they can either be manually covered by a cloth or masked in the software before performing the calibration.

After configuring the cameras, the calibration process starts with an empty scene where no markers should be detected, except for those attached to the calibration wand.

**Figure 6:**
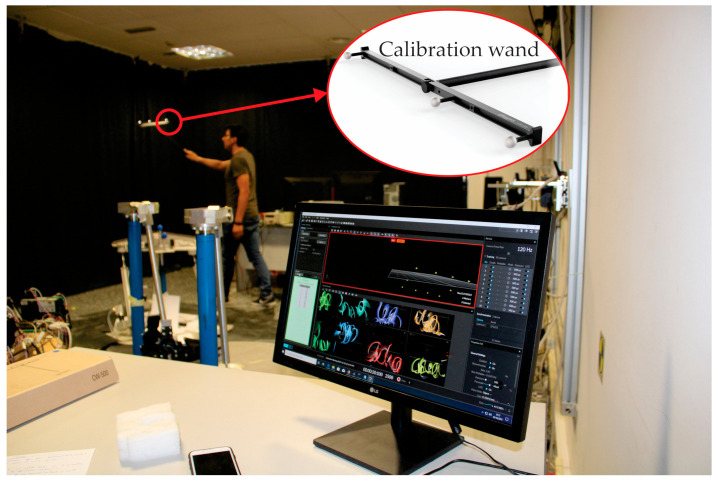
Calibration wand and experiment to determine the location of the markers.

By moving the calibration wand, which is provided by OptiTrack, around the workspace, the cameras provide successive 2D projections of the markers. The 2D projections are used to compute the relative position of the cameras. The software shows the increasing precision of this estimation as the process progresses (Figure 6), and when a high enough quality is achieved, the process is manually stopped.

**Figure 7:**
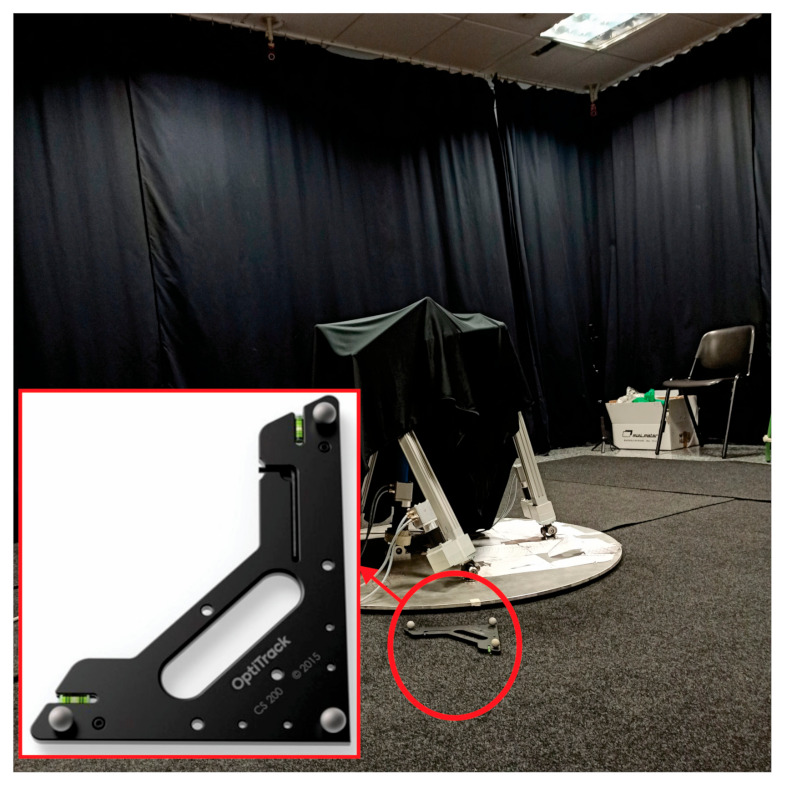
Calibration square.

The second tool, which concludes the calibration process, is the calibration square shown in Figure 7. This object includes three markers in right angle that define the origin and axes of the world coordinate system (also called ground plane by Motive). The ground plane is placed on the floor within the workspace area in such a way that its markers can be visible by as many cameras as possible. This tool incorporates a level to ensure its horizontal position.

Although all the cameras remain in the same position, minor movements of any of the cameras between experiments (for example, due to vibrations) can lead to poor tracking performance. For that reason, the calibration process must be performed once a day to ensure reliable 3D tracking. The calibration steps take no more than five minutes. After calibrating the cameras, Motive starts streaming data from all rigid bodies within the workspace. Rigid bodies are a set of 3 or more (maximum 20) markers whose relative distances remain constant. In this research, there are 2 rigid bodies represented by the fixed and mobile platform, respectively, and a set of 4 markers was attached to them. Three of the markers describe the cartesian coordinate frame of both platforms, and the fourth is added in a random (but known) position. If one of the markers is missed by the software during an experiment, the other three make it possible to reconstruct its position and keep streaming enough accurate data.

**Figure 8:**
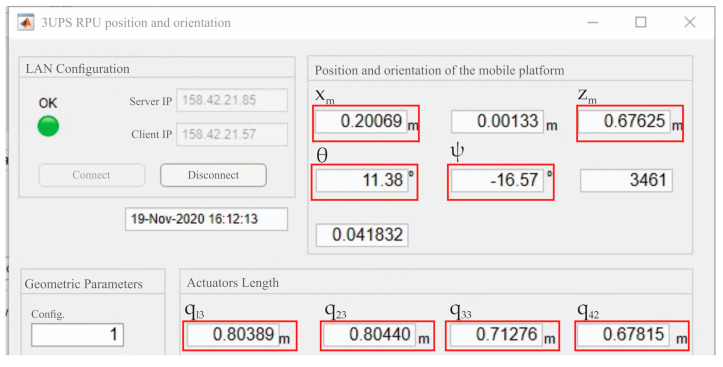
GUI for position and orientation tracking designed in MATLAB.

In the PR pose tracking App presented in this paper, the NatNet SDK provides a client class to communicate with the Motive server. A data handler is attached to this client, which works as a call-back that is executed every time there is a new frame of data available from the server. This handler has been used for retrieval of the x, y, z position of three markers placed on the fixed platform and another three placed on the mobile platform of the PR. Given the coordinates of the six markers, the actual position and orientation of the mobile platform (${\vec{X}}_{c}$) are calculated with respect to the $\left\{O_{f}-X_{F}Y_{F}Z_{F}\right\}\;$coordinate frame. Finally, ${\vec{X}}_{c}$ is sent through ROS2 messages to feed the control system. A MATLAB program has also been designed to provide an online view of ${\vec{X}}_{c}$ and calculate the actual actuator’s length by solving the inverse kinematics. Figure 8 presents the graphic user interface (GUI) for online measures of ${\vec{X}}_{c}$. It is important to note that this program is independent of the control system (and therefore runs in a personal computer) and simply offers a viewing tool.

### 2.5. Hybrid Controller Description

If a PR reaches a type II singularity, a controller must move the actuators to release the PR from the singularity, maintaining a minimum deviation from the original configuration. Therefore, a method to identify the best set of actuators to be moved is needed. The index $\Omega_{i,j}$, using the position and orientation of the mobile platform, is able to identify the actuators involved in the type II singularity. However, in a type II singularity, the measurement of the actual position and orientation of the PR require an external sensor, such as a 3DTS. For this reason, a novel controller able to release the PR under study from a type II singularity using the index $\Omega_{i,j}$ and a 3DTS is proposed. It is important to note that this is the first time that the index $\Omega_{i,j}$ is employed as an online proximity detector to a type II singularity.

**Figure 9:**
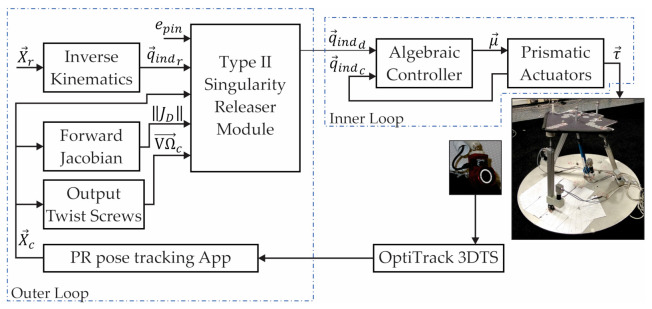
Hybrid controller architecture.

The novel vision-based hybrid controller to release the 3UPS+RPU PR from a type II singularity is shown in Figure 9. The hybrid controller combines two-level closed loops: an algebraic algorithm (inner loop) and a type II singularity releaser (outer loop). The type II singularity releaser calculates the $\Omega_{i,j}$ indices using the position and orientation of the PR provides by the OptiTrack 3DTS.

In the inner loop, the control signals ($\vec{\mu }$) to track the desired actuator’s location ${\vec{q}}_{{ind}_{d}}$ are calculated by an algebraic algorithm based on the measured location of the actuators ${\vec{q}}_{{ind}_{c}}$. The $\vec{\mu }$ is proportional to the forces ($\vec{\tau }$) applied by the linear actuators to move the mobile platform.

In the outer loop, the reference location of the actuators (${\vec{q}}_{{ind}_{r}}$) is obtained by solving the inverse kinematics for a knee rehabilitation trajectory (${\vec{X}}_{r}$), and ${\vec{X}}_{r}$ is designed for the 4-DOF of the 3UPS+RPU PR.

Based on the ${\vec{X}}_{c}$ measured by the 3DTS, the proximity to a type II singularity is detected by ${\vec{V\Omega }}_{c}$ and $||J_{D}||{}_{c}$ at every time step. ${\vec{V\Omega }}_{c}$ stores the six $\Omega_{i,j}$ indices as ${\left[\begin{array}{cc} \begin{array}{ccc} \Omega_{1,2} & \Omega_{1,3} & \Omega_{1,4} \end{array} & \begin{array}{ccc} \Omega_{2,3} & \Omega_{2,4} & \Omega_{3,4} \end{array} \end{array}\right]}^{T}$. If the 3UPS+RPU PR gets close to a type II singularity, ${\vec{q}}_{{ind}_{r}}$ is modified to define ${\vec{q}}_{{ind}_{d}}$. In Figure 9, the type II singularity releaser module (SRM) calculates the desired location of the actuator as follows:

(9) $${\vec{q}}_{{ind}_{d}}={\vec{q}}_{{ind}_{r}}+\nu_{d}\cdot t_{s}\cdot \vec{\Delta i}$$

where $\nu_{d}$ is the releasing velocity module for each actuator, $t_{s}$ stands for the controller sample time, and $\vec{\Delta i}$ represents an integer vector that counts the deviation required in the F actuators to release the PR from a type II singularity.

The SRM calculates ${\vec{q}}_{ind}{}_{d}$ at every time step, although $\vec{\Delta i}$ is modified only if an enable pin ($e_{pin}$) is activated. Two versions of the algorithms have been proposed to contrast the results when (i) moving the actuators that cause the singularity and (ii) moving the actuators that do not cause the singularity according to ${\vec{V\Omega }}_{c}$.

The first version (SRM-V1) releases a PR from a type II singularity by moving the limbs identified by ${\min\Omega }_{c}$, which represents the minimum value of ${\vec{V\Omega }}_{c}$. If ${\min\Omega }_{c}$ or $\|J_{D}\|{}_{c}$ is lower than a certain limit, $\Omega_{lim}$ and $\|J_{D}\|{}_{lim}$, respectively, the two rows of $\vec{\Delta i}$ that have to change are identified by ${\vec{i}}_{ch}$. The possible change combinations for the two rows of $\vec{\Delta i}$ are defined by the columns of $M_{inc}$ as follows:

(10) $$M_{inc}=\left[\begin{array}{cc} \begin{array}{cc} \begin{array}{cc} \begin{array}{c} 1\\ 1 \end{array} & \begin{array}{c} -1\\ -1 \end{array} \end{array} & \begin{array}{cc} \begin{array}{c} \;\;\;1\\ -1 \end{array} & \begin{array}{c} -1\\ \;\;\;1 \end{array} \end{array} \end{array} & \begin{array}{cc} \begin{array}{cc} \begin{array}{c} 1\\ 0 \end{array} & \begin{array}{c} -1\\ \;\;\;0 \end{array} \end{array} & \begin{array}{cc} \begin{array}{c} 0\\ 1 \end{array} & \begin{array}{c} \;\;\;0\\ -1 \end{array} \end{array} \end{array} \end{array}\right]$$

where 0, 1, and −1 correspond to the stop, unit forward motion, and unit backward motion commands for an actuator, respectively.

For each column of $M_{inc}$, an auxiliary variable ${\vec{q}}_{ch}$ is initialized as ${\vec{q}}_{{ind}_{d}}$, and then its elements indexed by ${\vec{i}}_{ch}$ are modified using the current $M_{inc}$ column. Then, it is checked that this position is confined within the geometrical limits. If ${\vec{q}}_{ch}$ is inside the actuators’ displacement range, the forward kinematic problem is solved (${\vec{X}}_{ch}$). Next, the angles reached by the spherical joints (${\vec{\alpha }}_{ch}$) are calculated. If ${\vec{\alpha }}_{ch}$ is within the working range, a new $\Omega_{i,j}$ is calculated for the limbs identified by ${\vec{i}}_{ch}$, and it is added to ${\vec{V\Omega }}_{ch}$. Then, $\vec{\Delta i}$ takes the value of the column of $M_{inc}$ that produces the maximum element of ${\vec{V\Omega }}_{ch}$ (${\max\Omega }_{ch}$), as that combination contributes the most to releasing the singularity without exceeding any range limit. Finally, ${\vec{q}}_{{ind}_{d}}$ is updated using the new $\vec{\Delta i}$.

An alternative algorithm called SRM-V2 has been proposed to test the behaviour when moving the wrong limbs. It modifies the rows of $\vec{\Delta i}$ that are not related to ${\min\Omega }_{c}$ (${\vec{i}}_{nc}$) to release the PR from the type II singularity caused by the actuators ${\vec{i}}_{ch}$. SRM-V2 is designed to verify that moving the actuators identified by ${\min\Omega }_{c}$ is the best way to release the 3UPS+RPU PR from a type II singularity.

The complete process performed by SRM-V1 is described in the pseudocode shown in Algorithm 1, where SRM-V2 is obtained by adding and replacing, the lines marked with * and **, respectively. A description of the variables used in Algorithm 1 is presented in Table 2.

**Algorithm 1.** Initialization 3***INITIALIZATION***                $\vec{\Delta i}=\vec{0}$ $N_{ch}=$ number of columns of $M_{inc}$. ***BEGIN*** ${\vec{q}}_{{ind}_{d}}={\vec{q}}_{{ind}_{r}}+\nu_{d}\cdot t_{s}\cdot \vec{\Delta i}$ **IF**
$e_{pin}==true$ ${\;\;\min\Omega }_{c}=$ minimum element in ${\vec{V\Omega }}_{c}$  **IF**
${\min\Omega }_{c}<\Omega_{lim}$ OR $\|J_{D}\|{}_{c}<\|J_{D}\|{}_{lim}$    **IF**
${\min\Omega }_{c}=={\vec{V\Omega }}_{c}\left(1\right)$      ${\vec{i}}_{ch}=\left[\begin{array}{cc} 1 & 2 \end{array}\right]$      $*{\;}^{\;}{\vec{i}}_{nc}=\left[\begin{array}{cc} 3 & 4 \end{array}\right]$    **ELSEIF**
${\min\Omega }_{c}=={\vec{V\Omega }}_{c}\left(2\right)$      ${\vec{i}}_{ch}=\left[\begin{array}{cc} 1 & 3 \end{array}\right]$      $*{\;}^{\;}{\vec{i}}_{nc}=\left[\begin{array}{cc} 2 & 4 \end{array}\right]$    **ELSEIF**
${\min\Omega }_{c}=={\vec{V\Omega }}_{c}\left(3\right)$      ${\vec{i}}_{ch}=\left[\begin{array}{cc} 1 & 4 \end{array}\right]$      $*{\;}^{\;}{\vec{i}}_{nc}=\left[\begin{array}{cc} 2 & 3 \end{array}\right]$    **ELSEIF**
${\min\Omega }_{c}=={\vec{V\Omega }}_{c}\left(4\right)$      ${\vec{i}}_{ch}=\left[\begin{array}{cc} 2 & 3 \end{array}\right]$      $*{\;}^{\;}{\vec{i}}_{nc}=\left[\begin{array}{cc} 1 & 4 \end{array}\right]$    **ELSEIF**
${\min\Omega }_{c}=={\vec{V\Omega }}_{c}\left(5\right)$      ${\vec{i}}_{ch}=\left[\begin{array}{cc} 2 & 4 \end{array}\right]$      $*{\;}^{\;}{\vec{i}}_{nc}=\left[\begin{array}{cc} 1 & 3 \end{array}\right]$    **ELSE**
${\min\Omega }_{c}=={\vec{V\Omega }}_{c}\left(6\right)$    ${\vec{i}}_{ch}=\left[\begin{array}{cc} 3 & 4 \end{array}\right]$      $*{\;}^{\;}{\vec{i}}_{nc}=\left[\begin{array}{cc} 1 & 2 \end{array}\right]$    **ENDIF**    ${\vec{V\Omega }}_{ch}=$ column vector of $N_{ch}$ zeros    **FOR**
$c_{1}=1:N_{ch}$      ${\vec{q}}_{ch}={\vec{q}}_{{ind}_{d}}$      ${\vec{q}}_{ch}\left({\vec{i}}_{ch}\right)={\vec{q}}_{ch}\left({\vec{i}}_{ch}\right)+\nu_{d}\cdot t_{s}\cdot M_{inc}\left(:,c_{1}\right)$      $**{\;}^{\;}{\vec{q}}_{ch}\left({\vec{i}}_{nc}\right)={\vec{q}}_{ch}\left({\vec{i}}_{nc}\right)+\nu_{d}\cdot t_{s}\cdot M_{inc}\left(:,c_{1}\right)$      **IF**
${\vec{minq}}_{ind}<{\vec{q}}_{ch}<{\vec{maxq}}_{ind}$ (element-wise comparison)        ${\vec{X}}_{ch}=$ Solve the Forward Kinematics for ${\vec{q}}_{ch}$, using ${\vec{X}}_{c}$ as initial condition        ${\vec{\alpha }}_{ch}=$ Angle of spherical joints for ${\vec{X}}_{ch}$        **IF**
${\vec{\alpha }}_{ch}<{\vec{\alpha }}_{lim}$ (element-wise comparison)            ${\vec{V\Omega }}_{ch}\left(c_{1}\right)=$ Calculate the index $\Omega_{i,j}$ for ${\vec{X}}_{ch}$ with $i,j={\vec{i}}_{ch}$
        **ENDIF**      **ENDIF**   **ENDFOR**    $c_{1}=argmax\left({\vec{V\Omega }}_{ch}\right)$    $\vec{\Delta i}\left({\vec{i}}_{ch}\right)=\vec{\Delta i}\left({\vec{i}}_{ch}\right)+M_{inc}\left(:,c_{1}\right)$    ${\vec{q}}_{{ind}_{d}}={\vec{q}}_{{ind}_{r}}+\nu_{d}\cdot t_{s}\cdot \vec{\Delta i}$  **ENDIF** **ENDIF** ***END***

Due to the properties of the index $\Omega_{i,j}$, the SRM algorithm has the advantage of moving a pair of F actuators simultaneously in each time step of the controller. For this reason, the SRM reduces the consumption of computing resources and the difference between ${\vec{q}}_{{ind}_{d}}$ and the original ${\vec{q}}_{{ind}_{r}}$.

## 3. Results

This section begins with a detailed description of the simulation setup, including the singular trajectories to be tested with SRM-V1 and SRM-V2 versions of the hybrid controller. Next, the performance of the hybrid controller in simulation is evaluated, where SRM-V1 appears to be better than SRM-V2. Subsequently, the experimental setup and the features of the actual 3UPS+RPU PR are detailed. Finally, the main experimental results show the effectiveness of the hybrid controller using SRM-V1 to release the PR under study from a type II singularity.

### 3.1. Simulation of the Vision-Based Hybrid Controller

Prior to implementing the algorithm on the actual PR, some simulations are performed on a kinematic and dynamic model of the 3UPS+RPU PR designed in MATLAB/Simulink. In both simulation and experimentation, the PR is moved from the initial position to a singular test configuration without activating the releaser. Then, it remains in the singular configuration for 15 s, after which the loop of the SRM is activated via $e_{pin}$. In that moment, one of the SRMs in Section 2.5 is launched based on the assumption that it will help release the robot from the type II singularity. The SRM launched has a lapse of 15s, allowing it to move the PR under study to a non-singular configuration.

Due to the lack of a simulated model of the 3DTS (see Figure 9) for MATLAB/Simulink, ${\vec{X}}_{c}$ is calculated directly by solving the forward kinematic problem. The main objective of the simulation is to test that the novel hybrid controller increases the values of $\left\|J_{D}\right\|$ and $\Omega_{i,j}$ in the vicinity of a type II singular configuration; i.e., it is able to release the PR under study from the type II singularity.

Since the 3UPS+RPU PR was built to interact with human knees, it is used to execute three rehabilitation movements: flexion of the hip, flexion–extension of the knee, and internal–external rotation of the knee [19]. This study, combining these three fundamental movements for simulation and experimentation, performs five knee rehabilitation trajectories ending in a type II singular configuration (see Table 3). The singular configurations of these five trajectories have $\left\|J_{D}\right\|$ and $\Omega_{i,j}$ close to zero but not exactly zero, avoiding several forward kinematic solutions in the simulation. All five knee trajectories are designed with constant velocity; in this case, the translational DOFs move at 0.02 m/s and the rotational ones at 0.03 rad/s.

**Figure 10:**
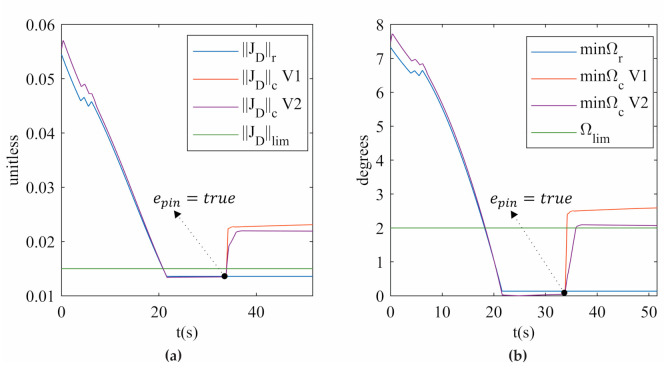
(**a**) $\left\|J_{D}\right\|$ (**b**) minΩ for trajectory 1 in the simulation.

The simulation of the five knee rehabilitation trajectories verifies that SRM-V1 and SRM-V2 release the 3UPS+RPU PR from a singular configuration. Figure 10 shows how the type II singularity indices $\|J_{D}\|{}_{c}$ and ${\min\Omega }_{c}$ increase when $e_{pin}$ is activated for trajectory 1. These results verify from an analytical perspective that the hybrid controller proposed releases the 3UPS+RPU PR from a type II singularity.

The performance of the proposed hybrid controller in tracking ${\vec{q}}_{ind}{}_{r}$ is evaluated by three overall measures:

- The mean absolute error (MAE)
  (11) $$\mathrm{MAE}=\frac{1}{F}\overset{F}{\underset{i=1}{\sum }}\left(\frac{1}{n}\overset{n}{\underset{j=1}{\sum }}\left|q_{ind}{}_{r}\left(i,j\right)-q_{ind}{}_{c}\left(i,j\right)\right|\right)$$
- The mean absolute percentage error (MAPE)
  (12) $$\mathrm{MAPE}=\frac{100}{F}\overset{F}{\underset{i=1}{\sum }}\left(\frac{1}{n}\overset{n}{\underset{j=1}{\sum }}\left|\frac{q_{ind}{}_{r}\left(i,j\right)-q_{ind}{}_{c}\left(i,j\right)}{q_{ind}{}_{r}\left(i,j\right)}\right|\right)$$
- The mean distance travelled for type II singularity release (MDSR)
  (13) $$\mathrm{MDSR}=\frac{1}{F}\overset{F}{\underset{i=1}{\sum }}\left(\overset{n}{\underset{j=1}{\sum }}\left|q_{ind}{}_{r}\left(i,k\right)-q_{ind}{}_{c}\left(i,j\right)\right|\right)$$
  where n is the number of samples taken after the activation of $e_{pin}$ at instant k, and i and j are the actuator and the time instant, respectively.

Table 4 shows the MAE, MAPE, and MDSR results for the simulation of the hybrid controller with SRM-V1 and SRM-V2. In this table, the MAE and the MAPE show that SRM-V1 has less error in position tracking than that of SRM-V2 during release from the type II singularity. In addition, the MDSR shows that SRM-V1 needs fewer movements of the actuators than SRM-V2 to release the PR from a singular configuration. These results show that moving the pair of actuators identified by the index $\Omega_{i,j}$ (SRM-V1) is the best option to release a PR from a type II singularity.

### 3.2. Experimentation of the Vision-Based Hybrid Controller

After testing the novel vision-based controller in simulation, the next step is implementing the hybrid controller on the real robot according to the diagram shown in Figure 9. Although both the simulation and experimentation have the same procedure, the experimentation presents two notable differences:

- ${\vec{X}}_{c}$ is provided by processing the data stream from the 3DTS in real time.
- During the 15 s before the SRM is activated, an external perturbation is applied to the PR. Since in a type II Singularity the PR can vary its position and orientation without moving any actuators, the researcher can apply some forces to the PR by hand to check whether the mobile platform experiences uncontrolled motion.

In the experimental context, the type II singularity release can be tested by trying to move the PR by hand before (when the PR is expected to move) and after the SRM is activated. After the activation of the SRM, the 3UPS+RPU PR will regain the stiffness required to ensure safe interaction with a patient.

Regarding the actual robot, the external limbs are driven by Festo DNCE 32-BS10 prismatic actuators, and the central limb is driven by a NIASA M100-F16 prismatic actuator. All the actuators are attached to Maxon 148867 150 W DC motors commanded by ESCON 50/5 servo controllers, which control the current by means of pulse width modulation (PWM). The current is proportional to the applied voltage (which comes from the control actions), and the torque is in turn proportional to the current. The DC motors are equipped with incremental encoders with a resolution of 500 counts per turn.

The control unit is connected to an industrial computer using acquisition cards. A PCI 1784 Advantech card is used to read the position from the encoders, having four 32-bit quadruple AB phase encoder counters. On the other hand, a 12-bit, 4-channel PCI 1720 Advantech card is used to send the control actions $\vec{\mu }$.

The proposed vision-based hybrid controller runs on the Robot Operating System 2 (ROS2) [31,32]. The two levels of the hybrid controller and the processing of the data stream from the 3DTS are implemented in a modular way using the C++ and Python programming languages. The controller receives the set of references ${\vec{q}}_{{ind}_{r}}$ from the solution of the inverse kinematics given the Cartesian references for the end-effector. The ${\vec{q}}_{{ind}_{r}}$ is sampled at a rate of 100 Hz, and the desired releasing velocity $\nu_{d}$ is set to 0.01 ms. These parameters are suitable for knee rehabilitation requirements.

For the actual PR, a fourth performance index is added to evaluate the smoothness of the movements performed by the controller, which is measured with the absolute variation rate (AVR) of the control actions as follows:

(14) $$\mathrm{AVR}=\frac{1}{F}\overset{F}{\underset{i=1}{\sum }}\left(\overset{n}{\underset{j=2}{\sum }}\left|\tau \left(i,j\right)-\tau \left(i,j-1\right)\right|\right)$$

During the first run of trajectory 1 using the hybrid controller with SRM-V2, the actual 3UPS+RPU PR reaches an AVR of 8N, which is too high for knee rehabilitation. For this reason, the experiment on the actual PR under study only focuses on the hybrid controller with SRM-V1. This decision is also supported by the better performance shown in the simulation (see Table 4).

Table 5 shows the results of performance tracking of ${\vec{q}}_{{ind}_{r}}$ of the hybrid controller with SRM-V1 implemented on the 3UPS+RPU PR. The MAE and MAPE for experimentation are similar to the simulation results, with a low AVR ensuring smooth movements of the mobile platform. In contrast, the actual MDSR is lower than the values calculated in the simulation. The reduction in MDSR is due to the accurate measure of ${\vec{X}}_{c}$ provided by the 3DTS, which is fundamental for a proper measure of the proximity to a type II singularity.

**Figure 11:**
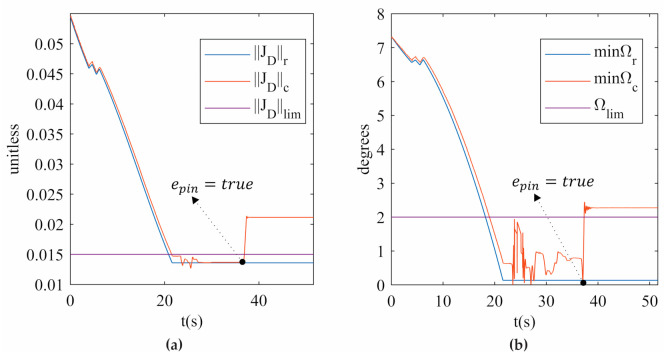
(**a**) $\left\|J_{D}\right\|$ (**b**) minΩ for trajectory 1 in the experimentation.

Figure 11 shows the measures of the two indices ($\|J_{D}\|{}_{c}$ and ${\min\Omega }_{c}$) when the actual PR is released from a singular configuration, corresponding to trajectory 1 with $\Omega_{3,4}$ as ${\min\Omega }_{c}$. The variation of $\|J_{D}\|{}_{c}$ and ${\min\Omega }_{c}$ before SRM-V1 is activated is due to the external force applied to the actual PR. It is important to mention that the actual PR recovers its stiffness at the end of all experiments. To the best of the author’s knowledge, this is the first time that an actual PR has been driven to a type II singularity and successfully released from it by using the index$\;\Omega_{i,j}$. The results can be seen in Video 1 and Video 2 provided as Supplementary Materials of this research.

**Figure 12:**
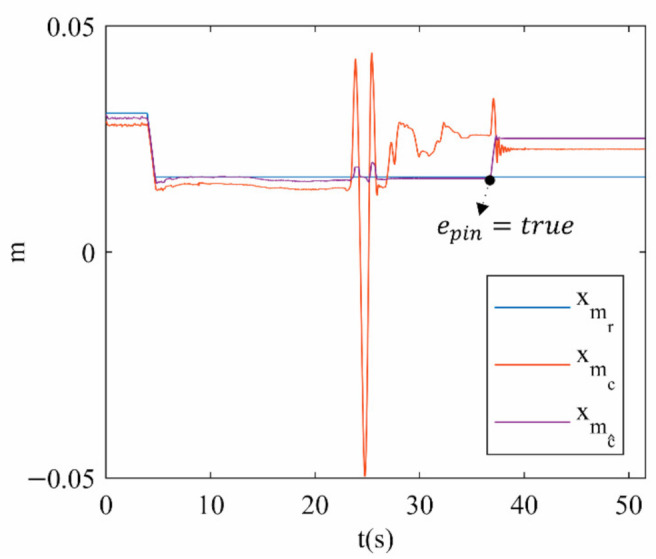
$x_{m}$ position for trajectory 1.

Figure 12 shows the reference (r) trajectory for $x_{m}$ in contrast to its estimation ($\hat{c}$) by using the forward kinematic model and the experimental measures (c) based on data streaming from the 3DTS. Despite both estimated and experimental measures being calculated online, only the experimental measure detects the movement produced by the external force applied to the PR. This verifies that when the 3UPS+RPU PR is in a type II singularity, the actual $x_{m}$ cannot be determined by solving the forward kinematic.

**Figure 13:**
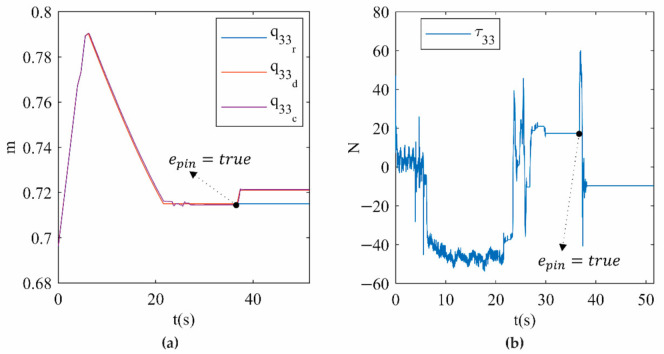
(**a**) $q_{ind}$ (**b**) τ on limb 3 for trajectory 1.

Figure 13a shows the position for limb 3, which is one of the two limbs involved in the type II singularity in trajectory 1. In this figure, the measured position (c) accurately tracks the desired position (d), which differs from the reference (r) only after SRM-V1 activation. Furthermore, Figure 13a clearly shows that the desired position is modified by a few millimetres from the reference to release the actual PR from the type II singularity. Finally, Figure 13b shows the smooth control actions calculated by the hybrid controller implemented on the actual PR using SMR-V1. Video 1 provides an interactive view of the results presented in Figure 12 and Figure 13 and can be found in the Supplementary Materials Section.

The experimental results conclude that the vision-based hybrid controller with SMR-V1 releases an actual PR from a type II singularity with minimum deviation from the original reference. In addition, the OptiTrack 3DTS allows the hybrid controller with SMR-V1 to take advantage of the features of the index $\Omega_{i,j}$.

## 4. Discussion

This study has addressed the novel task of releasing a 4-DOF PR from type II singular configuration using the index $\Omega_{i,j}$ to identify the limbs involved in the singularity. The hybrid controller proposed combines an algebraic controller with an external computational loop that modifies the joint references only for the limbs that are causing the singularity. This mechanism can be activated whenever the robot enters into a type II singularity by measuring the $\|J_{D}\|{}_{c}$ and ${\min\Omega }_{c}$. Both $\|J_{D}\|{}_{c}$ and ${\min\Omega }_{c}$ are measured based on the actual position and orientation of the mobile platform that is provided online by a OptiTrack 3DTS. The embedded sensorization includes a set of encoders attached to the motors to ascertain the joint positions.

To show the effectiveness of the designed method, several experiments have been conducted with trajectories that leave the robot in distinct singular configurations, where the releasing algorithm is activated. This scheme has been implemented in both simulation and actual settings to compare the differences in performance when moving the limbs involved (SRM-V1) or not involved (SRM-V2) in the type II singularity. The algorithm for SRM-V1 and SRM-V2 defined the movement of the actuators based on the results of ${\min\Omega }_{c}$.

The results of the simulation in Section 3.1 clearly show that SRM-V1 makes the robot behave better in terms of all the performance measures with respect to SRM-V2. According to Table 4, SRM-V1 presents a 0.54% (4.09 mm) mean error in tracking the original reference with a mean distance travelled of 7.95 mm for releasing the PR from a type II singularity. These errors are approximately half of those obtained with SRM-V2, thus verifying that moving the actuators identified by ${\min\Omega }_{c}$ is the best option to release the 3UPS+RPU PR from a type II singularity. In fact, no trajectories were performed with the actual robot using SRM-V2, as a first experiment using this algorithm showed that the robot was struggling to get out of the singular configuration, with sharper control actions than those obtained in simulation.

Section 3.2 shows that by using knowledge of the true position and orientation of the mobile platform, the hybrid controller with SRM-V1 can successfully release the actual PR from a type II singularity. All singular trajectories were overcome, even in the cases where the mobile platform was manipulated to change its position during the standby time. The results show how the simulated and real experiments are alike, as all of the indicators for SMR-V1 are somewhat similar. These errors are proven to be dependent on the starting singular configuration, since trajectory 5 is harder for the PR to overcome.

Based on the results of simulation and experimentation, this is the first use of a vision-based hybrid controller capable of releasing a 4-DOF PR from a singular configuration. It is also notable that the effectiveness of the release from a type II singularity with a minimum deviation of the original reference depends on ${\min\Omega }_{c}$. The smoother response of the vision-based hybrid controller is achieved because of the accurate measures of the 3DTS, making it a fundamental element of the hybrid controller. It is important to highlight that before this research, the $\Omega_{i,j}$ had not been used as an online detector of the proximity to type II singularities for controlling purposes.

The proposed vision-base hybrid controller compensates a main drawback of PRs, and it represents a step forward towards compliant manipulation of PRs. This system improves the performance of knee rehabilitation tasks by ensuring the safety of the patient during human–robot interaction, even if the PRs arise a type II singularity.

In future research, SRM-V1 can be extended for its use in the task of type II singularity avoidance, i.e., preventing the PR from entering into a singular configuration. Although little literature exists regarding this field, the SRM-V1 algorithm offers valuable insight into the limbs that are expected to lead the robot to a type II singularity. After adding the possibility of returning to the original reference to SRM-V1, the avoidance of type II singularities could be achieved in a more reliable way than using other methods such as artificial potential fields.

## Figures and Tables

**Table 1:** Geometric parameters for the 3UPS+RPU PR.

$R_{1}\;\left(m\right)$	$R_{2}\;\left(m\right)$	$R_{3}\;\left(m\right)$	$\;\beta_{FD}\;\left({}^{\circ}\right)$	$\beta_{FI}\;\left({}^{\circ}\right)$	$d_{s}\;\left(m\right)$
0.4	0.4	0.4	90	45	0.15
$R_{m1}\;\left(m\right)$	$R_{m2}\;\left(m\right)$	$R_{m3}\;\left(m\right)$	$\;\beta_{MD}\;\left({}^{\circ}\right)$	$\beta_{MI}\;\left({}^{\circ}\right)$	
0.3	0.3	0.3	50	90	

**Table 2:** Description of parameters, inputs, and outputs of SRM-V1 and SRM-V2.

Parameters		
Variable	Description	Default
$\nu_{d}$	releasing velocity module in m/s	0.01
$t_{s}$	controller sample time in s	0.01
$\|J_{D}\|{}_{lim}$	$\mathrm{experimental}\;\mathrm{limit}\;\mathrm{for}\;\|J_{D}\|$	0.015
$\Omega_{lim}$	$\mathrm{experimental}\;\mathrm{limit}\;\mathrm{for}\;\Omega_{i,j}$	1.8°
${\vec{maxq}}_{ind}$	maximum feasible values for the actuators’ length in m, 4x1 vector	${\left[\begin{array}{cc} \begin{array}{cc} 0.93 & 0.92 \end{array} & \begin{array}{cc} 0.93 & 0.82 \end{array} \end{array}\right]}^{T}$
${\vec{minq}}_{ind}$	minimum feasible values for the actuators’ length in m, 4x1 vector	${\left[\begin{array}{cc} \begin{array}{cc} 0.65 & 0.64 \end{array} & \begin{array}{cc} 0.65 & 0.54 \end{array} \end{array}\right]}^{T}$
${\vec{\alpha }}_{lim}$	experimental limits for the spherical joints, 3x1 vector	38°
$M_{inc}$	$\mathrm{possible}\;\mathrm{increments}/\mathrm{decrements}\;\mathrm{for}\;\vec{\Delta i}$	See equation (10)
$\vec{\Delta i}$	column vector 4x1, persistent variable	-
**Inputs**		
**Variable**	**Description**	**Default**
$e_{pin}$	enable pin	-
$\|J_{D}\|{}_{c}$	determinant of the forward Jacobian matrix, feedback signal	-
${\vec{V\Omega }}_{c}$	column vector with the six $\Omega_{i,j}$ indices, feedback signals	-
${\vec{X}}_{c}$	position and orientation of the mobile platform, feedback signal	-
${\vec{q}}_{{ind}_{r}}$	trajectory for the actuators, reference signal	-
**Outputs**		
**Variable**	**Description**	**Default**
${\vec{q}}_{{ind}_{d}}$	trajectory for the actuators, desired signal	-

**Table 3:** Description of the trajectories with a type II singularity at the end.

Trajectory	Description	Type II Singularity			
		$x_{m}\;(m)$	$z_{m}\;(m)$	θ (rad)	ψ (rad)
1	Hip flexion	0.01	0.70	0.15	0.31
2	Partial internal–external knee rotation	0.01	0.70	−0.02	0.14
3	Flexion–extension of the knee combined with ankle and knee rotations	0.05	0.72	−0.01	0.15
4	Flexion–extension of the knee combined with hip flexion	0.12	0.77	−0.06	0.11
5	Complete internal–external knee rotation	−0.05	0.73	0.10	0.33

**Table 4:** Performance of the hybrid controller using SRM-V1 and SRM-V2 in the simulation.

Trajectory	MAE (mm)		MAPE (%)		MDSR (mm)	
	SRM-V1	SRM-V2	SRM-V1	SRM-V2	SRM-V1	SRM-V2
1	3.87	10.74	0.53	1.40	7.01	18.18
2	1.09	2.04	0.14	0.28	5.05	2.92
3	1.77	6.15	0.24	0.82	4.78	6.74
4	3.00	10.24	0.38	1.25	7.48	10.81
5	10.74	10.44	1.43	1.37	15.47	35.23
***MEAN***	**4.09**	**7.92**	**0.54**	**1.02**	**7.95**	**14.77**

**Table 5:** Performance of the hybrid controller using SRM-V1 in the experimentation.

Trajectory	MAE (mm)	MAPE (%)	MDSR (mm)	AVR (N)
1	3.26	0.45	3.64	0.22
2	3.02	0.41	7.61	0.52
3	2.05	0.27	1.60	0.17
4	2.14	0.27	1.90	0.46
5	10.66	1.42	11.82	1.44
***MEAN***	**4.22**	**0.56**	**5.31**	**0.56**

## Data Availability

Not applicable.

## Supplementary Materials

The following videos are available online at https://imbio3r.ai2.upv.es/videos/TypeII_singularities: Video 1: 4-DOF parallel robot: vision-based hybrid controller to release from a type II singularity. Trajectory 1; Video 2: 4-DOF parallel robot: vision-based hybrid controller to release from a type II singularity. Trajectory 5.
